# ‘The Laryngectomee Guide’ in Malaysian Language

**DOI:** 10.21315/mjms2021.28.3.17

**Published:** 2021-06-30

**Authors:** Itzhak Brook

**Affiliations:** Georgetown University School of Medicine, Washington DC, USA

**Keywords:** laryngectomee, speech, rehabilitation, radiation, chemotherapy

## Abstract

‘The Laryngectomee Guide’ (*Panduan Pesakit Laringektomi*) is available now in Malaysian language. The Guide provides information that can assist laryngectomees and their medical providers about medical, dental and psychological issues. It contains information about side effects of radiation and chemotherapy; methods of speaking; airway, stoma, and voice prosthesis care; eating and swallowing; medical, dental and psychological concerns; respiration; anesthesia; travelling and how to cope with the COVID-19 pandemic.

Dear Editor,

I am happy to announce that the ‘The Laryngectomee Guide’ (*Panduan Pesakit Laringektomi*) is available now in Malaysia as an eBook (free) and paperback. The translation was done by Professor Dr Marian Mat Baki, Professor Dr Mohd Razif Mohamad Yunus and Associate Professor Dr Marwaddah Azman from Universiti Kebangsaan; and Professor Dr Primuharsa Putra Sabir Husin Athar from KPJ University College, Kuala Lumpur, Malaysia.

Paperback is available at http://bit.ly/3n4fK3m and eBook (free) at http://bit.ly/2X29Rcu

The Guide provides information that can assist laryngectomees and their medical providers about medical, dental and psychological issues. It contains information about side effects of radiation and chemotherapy; methods of speaking; airway, stoma, and voice prosthesis care; eating and swallowing; medical, dental and psychological concerns; respiration; anesthesia; travelling and how to cope with the COVID-19 pandemic.

The American Academy of Otolaryngology Head and Neck Surgery made the English edition available for free download on their website: http://www.entnet.org/content/laryngectomee-guide

The laryngectomee guide is also available in English, traditional and simple Chinese, Japanese, Korean, Filipino, Indonesian, Russian, French, Romanian, Bulgarian, Turkish, Greek, Bosnian, Croatian, Arabic, Farsi, Spanish, Portuguese and Italian (http://dribrook.blogspot.com/2018/08/the-laryngectomee-guide-is-available-in.html).

I hope that the Guide will be helpful to laryngectomees and their medical providers in Malaysia.

